# The effect of the cultural formulation interview on therapeutic working alliance: a study protocol

**DOI:** 10.3389/fpsyt.2024.1322356

**Published:** 2024-03-04

**Authors:** Alma M. Brand, Simon P. N. Groen, Nathalie Destoop, Hannah E. Jongsma, Samrad Ghane, Bernard G. C. Sabbe, Harry van Velsen, Kurt van Houten, Özlem Becan, Dhiya Al Alyan, Mario H. Braakman

**Affiliations:** ^1^ Tilburg Law School, Tilburg University, Tilburg, Netherlands; ^2^ De Evenaar, Center for Transcultural Psychiatry, GGZ Drenthe, Beilen, Netherlands; ^3^ Mobile 2B Team SPITT and Culture-sensitive Care POZAH, Psychiatric Hospital Sint-Alexius Grimbergen, Brussels, Belgium; ^4^ Center for Transcultural Psychiatry Veldzicht, Balkbrug, Netherlands; ^5^ University Center of Psychiatry, University Medical Center Groningen (UMCG), Groningen, Netherlands; ^6^ Parnassia Group, Utrecht, Netherlands; ^7^ Faculty of Medicine and Health Sciences, Collaborative Antwerp Psychiatric Research Institute, University of Antwerpen, Antwerpen, Belgium; ^8^ Consultant, Groningen, Netherlands

**Keywords:** cultural formulation interview (CFI), perceived cultural empathy, empathy, therapeutic working alliance, RCT, migrant patients

## Abstract

**Background:**

The Cultural Formulation Interview (CFI) is designed to improve understanding of patients’ mental health care needs. The lack of empirical evidence on the impact and effectiveness of CFI use in clarifying people’s perspectives, experiences, context, and identity, and in preventing cultural misunderstandings between migrant patients and clinicians, inspired this study. The objective is to examine the effect of the CFI on the strength of therapeutic working alliances, and the potential mediating or moderating role of perceived empathy.

**Materials and methods:**

A multicenter randomized controlled trial will be conducted, involving migrant patients, their confidants, and clinicians. The CFI will be administered in the intervention group, but not in the control group. Validated questionnaires will be used to assess therapeutic working alliances and perceived empathy. T-tests and linear regression analyses will be conducted to investigate between-group differences and possible mediating or moderating effects.

**Results:**

This study will indicate whether or not the CFI strengthens the therapeutic working alliance between patients and clinicians, as moderated and/or mediated by perceived empathy.

**Discussion:**

Research on the effect and impact of using the CFI in mental health care for migrant patients is important to clarify whether its use strengthens the therapeutic working alliance with clinicians. This can lead to a reduction in cultural misunderstandings and improve mental health care for migrant patients. The results may also be important for the implementation of the CFI as a standard of care.

**Ethics and dissemination:**

This research protocol was tailored to the needs of patients in collaboration with experts by experience. It was approved by the Ethical Review Board of the Tilburg Law School and registered in the Clinical Trials Register under number NCT05788315. Positive results may stimulate further implementation of the CFI in clinical practice, and contribute to improving the impact of the CFI on the therapeutic working alliances.

## Introduction

Due to cultural differences between patients with a migrant background (hereafter: migrant patients) and clinicians, mental health care faces many challenges. Such differences cause higher thresholds for help-seeking and incongruencies in diagnosis and treatment, which do not meet these patients’ needs (1). Challenges stem from language barriers, diverse idioms of distress, and distinct explanatory models between migrant patients and predominantly non-migrant mental health providers (2). Idioms or expressions of distress provide alternate means of conveying emotional upset and are indicative of personal and cultural contexts. In mental health care, migrant patients experience higher dropout rates, longer care trajectories, lower treatment effectiveness, and more frequent involuntarily admissions to clinical care (3, 4). Despite the positive fact that migrant patients’ access to mental health care appears to have improved, they often do not receive the necessary care (5). This could result from a limited view of these patients experiencing mental health symptoms and inadequate focus on their social context, cultural roots, environment, and cultural identity, which fails to recognize and comprehend the individual behind the patient (6, 7). Despite the consideration of migrant patients’ cultural identities in diagnostic procedures to gain better insights into their symptoms, these patients continue to receive inadequate diagnoses and often feel misunderstood (1, 6, 7).

To address miscommunication in diagnosis and treatment, initiatives have been taken to include cultural identity, idioms of distress, support systems, and clinician-patient relationships in interviews (1, 8). In 2013, the Cultural Formulation Interview (CFI) was included in the fifth edition of the Diagnostic and Statistical Manual of Mental Disorders (DSM-5) (9). This semi-structured interview incorporates the migrant patient’s social and cultural context and addresses beliefs, customs, and identity in the diagnostic and treatment process. The interview aims to increase mutual understanding and rapport between patients and clinicians based on an interest in and a better understanding of the cultural background of these patients to prevent cultural misunderstandings (10). The CFI was found to be a feasible, acceptable, and useful instrument in mental healthcare and it is used increasingly among different populations worldwide (11–13). In general, therapeutic working alliances benefit from the use of the CFI (7, 14). However, CFI use can also negatively affect communication and the therapeutic working alliance between migrant patients and clinicians when questions about culture may upset the patients and jeopardize the quality of the alliance (7, 15, 16). To avoid the latter scenario, it is recommended to train clinicians in the use of the CFI, provide supervision, and focus on compliance and fidelity in administering the interviews (7). It is still unclear whether this strategy creates stronger therapeutic working alliances, which may contribute to reducing high dropout rates, improving treatment outcomes, and enhancing the quality of mental health care (7).

One of the most important factors in mutual understanding between migrant patients and clinicians is empathy. Empathetic clinicians will better understand their migrant patients than non-empathic clinicians (17). Empathy is described as the accurate recognition of the internal frame of reference of a person other than oneself, integrated with the emotional components and implications of imagining oneself as the other person (18). Empathy is part of building relationships between patients and clinicians (19). Empathy may be even more important in culturally sensitive mental health care. Cultural empathy embodies the ability to identify with the feelings, thoughts, and behaviors of people from cultural backgrounds different from one’s own by listening and hearing beyond the spoken word and bridging cultural differences (20).

Evidence of the effectiveness of the CFI in clinical practice is still lacking (6). An exploratory qualitative study among migrant patients found tentative optimistic signs regarding satisfaction, recognition, and clarity of the effect of cultural formulation (21). However, resistance to CFI implementation was also reported from both migrant patient and clinician perspectives. This resistance was based on the experience of some problematic CFI questions (7). The present study aims to clarify whether the CFI leads to stronger therapeutic working alliances and if this association is mediated and/or moderated by higher perceived cultural empathy. The results may contribute to better suggestions for mental health assessments. If the use of the CFI is found to be beneficial to the therapeutic relationship, intake, diagnostic, and therapeutic protocols in clinical mental health care could be adapted and improved. In addition, the timing of administration of a CFI could be optimized and protocolized, including experiential learning in intake, diagnostic, and treatment procedures.

It is hypothesized that this study will show that implementation of the CFI may have a positive, strengthening effect on the therapeutic working alliance between migrant patients and clinicians moderated by (perceived) cultural empathy. Therefore, the central research question of this study is: Does the use of the CFI influence the therapeutic working alliance between migrant patients and clinicians? In addition, it will be examined whether perceived cultural empathy moderates (changes strength and direction) or mediates (explains) the effect of CFI use on the therapeutic working alliance.

## Methods and analyses

### Study design

This study has been designed as an RCT among migrant outpatients newly admitted to mental health care and their clinicians in four mental health care settings in the Netherlands. Experiential expertise and knowledge were crucial in this study design. Five experts by experience were recruited to help plan this research when providing their services at one of the participating locations. They contributed significantly to the study design. Four experts by experience will assist the researchers with recruitment and enrollment, which may lower the participation threshold for migrant patients. Their assistance in answering questions during the data collection period may lead to increased enrollment, adherence, and better outcomes. In addition, the experts will assist with the interpretation of the results based on their experiences and their expectations.

### Study context

There is a lack of scientific evidence regarding the effect of using the CFI on therapeutic working alliances. To the best of our knowledge, this study is the first to use an RCT to investigate this effect, including the exploration of the moderating or mediating effect of cultural empathy. To enhance the quality of this research, and acknowledge the growing importance of experiential expertise in mental health care, experts by experience were democratically involved in the development of this research protocol, the establishment of the research questions, highlighting the research topic from their perspective, and included as coauthors in this paper.

Funding for this study was provided by ZonMw under grant number 6390039231 based on the argument that the aim of this study is highly relevant for improving the quality of mental health care for patients with a migration background. Based on the results of the study, ZonMw advised contacting health insurance companies to support the implementation of the CFI as a standard care practice if this study yields significant results. This study will be conducted in accordance with the tenets of the Declaration of Helsinki (22). The study protocol was approved by the Ethical Review Board of Tilburg University under identification code TLS_RP978 and registered in the Clinical Trials Register under the number NCT05788315.

### Co-creation and user involvement

Experts by experience played an important role in determining the study parameters, based on their personal experiences with mental health symptoms and mental health care, such as stigma and empowerment. They know from experience how important it is to create a supportive environment for patients in need of mental health care and how this can enhance supportive recovery care (23–25). Their cultural background and personal experience with mental health symptoms, treatment, and recovery trajectories distinguish them from clinicians and researchers, highlighting the research topic from their perspective. The experts by experience have particularly emphasized the importance of incorporating empathy into the study design. Based on their practical experience and knowledge, they are able to understand patient’s associated feelings of vulnerability with mental health symptoms. This understanding allows migrant patients to feel safe and authentic during their interactions. In addition, they are able to empathize with migrant patients and recognize coping strategies to address mental health symptoms. Experts by experience also advised to include migrant patients’ confidants such as a partner, family member, close friend, or other support contacts to participate in this study to learn from their experiences with the migrant patient’s mental health. Their input could be beneficial to both the migrant patient and the clinician by clarifying the migrant patient’s circumstances from their perspectives. Therefore, the experts by experience will continue to contribute to the conduct of this study (17).

### Participants

Participants eligible for inclusion in this study are adults aged 18 and over who have a personal or family history of migration, are voluntarily admitted for outpatient mental health care, and are able to communicate their vision, context, and expectations for the treatment and care provided. Migrant patients were included regardless of their administrative status. In addition to the migrant patients, clinicians, and, where applicable, a migrant patient’s confidant will also participate in the study. Inpatients and patients who undergo compulsory treatment are excluded from participation to avoid bias through compromised therapeutic working alliances due to the complex nature of the mental health problems presented in inpatients and involuntary admissions. Participants are excluded when they suffer from acute mental illnesses, such as psychotic episodes, that prevent them from adequate participation.

In Dutch mental health centers, care is provided by multidisciplinary teams of clinicians consisting of psychiatrists, psychologists, psychiatric nurses, social workers, and various types of specialist therapists. Some mental health centers have specialized transcultural teams that were recruited for participation in this study. Participating clinicians must be willing to adhere to the study protocol. The participating Dutch mental health centers are the Center for Transcultural Psychiatry Veldzicht, ProPersona, Parnassia, and GGZ Drenthe. Each center will try to include a representative group of participants in this study. The aim is to have an equal number of participants in the intervention and control groups in each participating center. Data collection will take place between June 2023 and June 2024.

### Procedure

**Figure 1:**
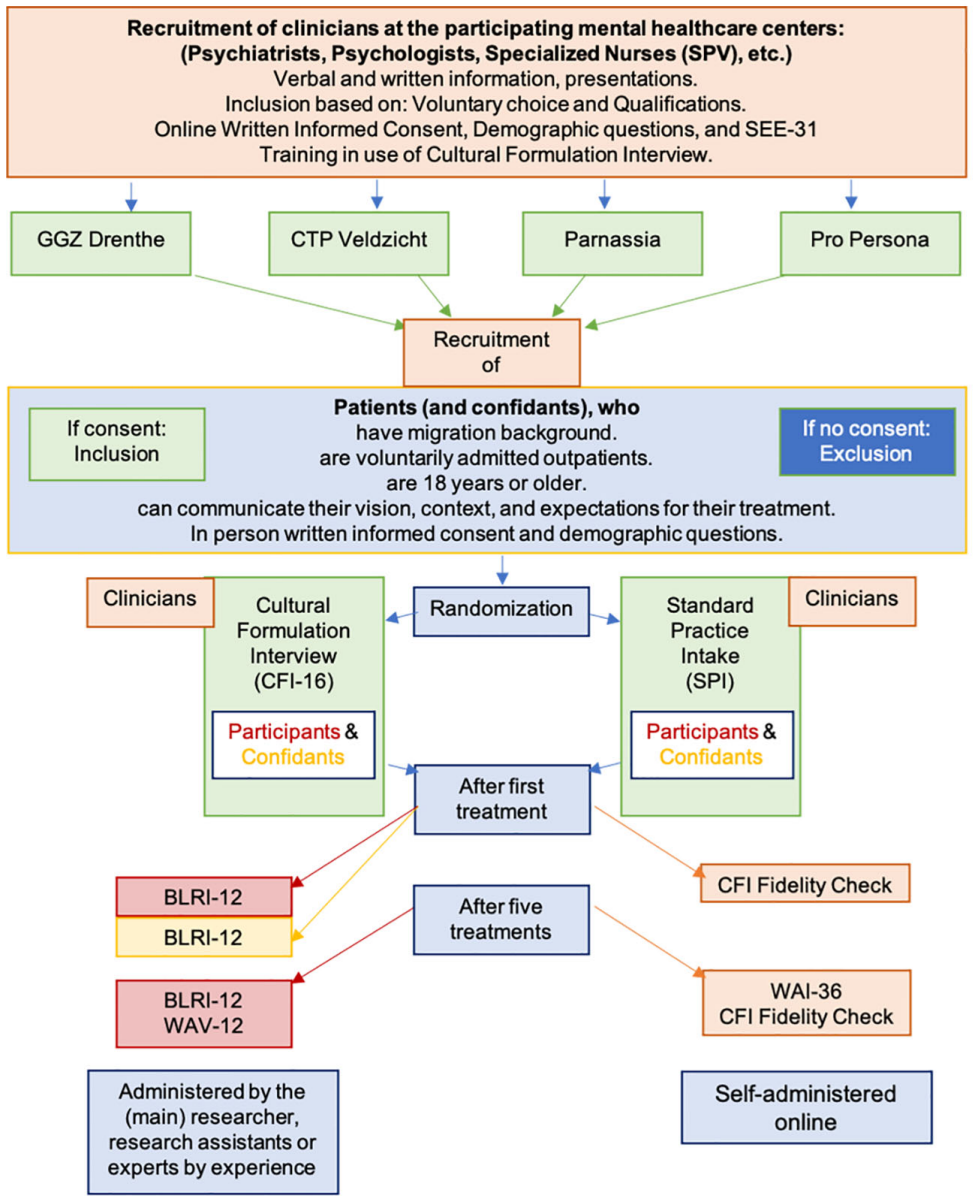
The Procedure of Cultural Formulation Interview Randomized Controlled Trial.

**Figure 1** illustrates the recruitment and inclusion procedure in this study. Before migrant patient enrollment, clinicians at each mental health center will receive an information letter about the study. They will also receive a link to an online informed consent form and some demographic questions. Subsequently, all participating clinicians will be instructed on how to apply the inclusion criteria for ongoing recruitment and will be trained to administer the CFI to increase the level of adherence to the intervention and study protocol. Therefore, clinicians will be invited to participate in a two-hour training session for the CFI at their site. The session includes a review of the core guidelines, a 24-minute video demonstration, interactive behavioral simulations with coaching and feedback from local principal researchers, and a question-and-answer session. All clinicians at participating centers are encouraged to follow the interview structure throughout the study to ensure consistency regardless of the status of the migrant. During the term of the study, the researchers will schedule at least two, or more if necessary or requested, evaluation moments with the clinicians to assess the recruitment of participants, the administration of the CFI based on fidelity checks, and personal experiences with the study protocol. The experts by experience will be contact persons for each of the centers and will participate in the CFI training and instruction at the center to which they were assigned.

### Recruitment of migrant patients and confidants

Clinicians will recruit migrant patient participants during their intake sessions. They will provide brief information about the study, and hand out an information letter on behalf of the researchers. The information letter is made available in several languages, including Dutch, English, Spanish, French, Turkish, classic Arabic, and Farsi. Translations have been done and/or reviewed by at least two native speakers of each language. Clinicians will be instructed to ask the potential participants for permission to provide their name, email address, and/or telephone number to the principal investigator in a secure online environment, with permission to be contacted by the principal investigator after one week regarding participation. During this follow-up, any questions about participation will be answered, information clarified, and, if the patient agrees to participate, an appointment will be made to sign the informed consent form and complete some demographic questions. Additionally and when applicable, contact information for a confidant who is willing to participate will be collected, and confidants will be contacted using the same process as the migrant patients. The informant version of the CFI (CFI-I) will be administered by the migrant patient’s clinician in accordance with the patient’s randomization. Clinicians and confidants will also have to sign an informed consent form before participation. Participation is voluntary, does not jeopardize treatment, and participants are free to withdraw their consent to participate at any time without explanation or negative consequences. The researchers or clinicians may decide to withdraw a patient from the study for urgent medical reasons. The procedure of this study is shown in **Figure 1**.

### Randomization

After enrollment, the migrant patients will be randomly assigned to the intervention or the control group by the principal investigator. During the data collection period, the data will not be fully anonymous, because the principal investigator needs to be able to plan appointments for data collection with both the migrant patients and clinicians and to avoid the risk of making mistakes in assigning the collected data to the correct participant ID in the main data file. Patients are single-blinded while their clinicians will be notified of the randomization status to use the CFI or adhere to standard care. Randomization will be set up based on the output of the online tool, Research Randomizer (26). Four groups, representing the four mental health centers and 100 participants per center, are entered into the tool. The resulting four sets of random order numbers, up to 100, are stored and used to randomize participants. Set 1 is used for GGZ Drenthe, 2 for Parnassia, 3 for CTP Veldzicht, and 4 for ProPersona. Odd numbers indicate the intervention group and even numbers indicate the control group. The order of the numbers defines the allocation to the respective participants in the order of inclusion. The same numbers are also used as ID numbers for the participants. The letters D, U, V, and P are added to the ID numbers to indicate the mental health center; D for GGZ Drenthe, U for Parnassia, V for CTP Veldzicht, and P for ProPersona. In addition, the letters MP for the main participant, C for clinician, and Co for confidants are added to the numbers to identify the role of the participants in the study. In this way, the number and treatment center letter link the participant to their clinician and confidant, making them anonymous but identifiable in their association. Before data analyses, the data file will be fully anonymized and the coding system changed by non-analyzing researchers to blind the researchers performing the analyses from knowing which participant was allocated to the intervention or the control group, and in which mental healthcare center they participated.

### Data collection

For the duration of the study, migrant patients will be assigned to a personal clinician as they will be required to complete questionnaires about each other. Before the start of treatment, clinicians will be informed of the randomization group to which the migrant patient has been assigned. Participating confidants will have one consultation with the personal clinician of the patient that they represent, according to the patient’s randomization status.

The principal investigator will receive notification regarding the date of the initial treatment session to schedule an appointment for the migrant patient to take the first-stage survey on perceived empathy, either face-to-face or online. The respective questionnaires must be completed within one week of the first session, and interpreters will be employed as necessary. The patient proceeds with subsequent treatment following the treatment plan. The procedure for participating confidants will be identical, and their involvement will cease upon finishing the perceived empathy questionnaire (see **Figure 1**).

In the intervention group, treatment sessions will proceed using CFI questions. The control group will receive standard care in which CFI questions are avoided. After each initial treatment session, clinicians will complete a brief online questionnaire to evaluate their fidelity to the CFI. Fidelity is expected for migrant patients who were randomized in the intervention group, and infidelity to the CFI is expected in the control group. In addition, the clinicians will be asked to respond to empathy-related questions.

The principal investigator will receive notice of the migrant patient’s fifth treatment session appointment with their personal clinician to schedule the second-stage questionnaire completion. The patients will have an in-person or online appointment where they will answer the same questions about perceived empathy, in addition to supplementary questions about the therapeutic working alliance. The questionnaires need to be completed within one week of the fifth session. After the completion of these questionnaires, the patient’s participation concludes. Subsequently, clinicians will conduct an online questionnaire after every fifth treatment session. This questionnaire includes the same fidelity check and empathy questions as offered after the first treatment session and additional questions regarding the therapeutic working alliance. Clinicians will continue treating other study participants and are required to reiterate the aforementioned protocol for each participating patient under their care. Their participation in this study will end after completing the questionnaires of their final migrant patient.

### Benefits and consequences of participation

In line with the aim of this study, patients in the intervention group may benefit from the experience of a stronger therapeutic working alliance and a higher level of perceived cultural empathy. The risk of negative consequences of participation is estimated to be as low, if not non-existent since patients in the control group also receive the current standard of treatment and care. The additional burden of participation for patients is to complete questionnaires before and after the first and after the fifth treatment session, which will take some extra time.

Participating clinicians benefit from free CFI training that can enhance their clinical competencies and future therapeutic working alliances with patients and their confidants. The additional obligation for participating clinicians entails investing additional time in consultations with confidants and completing the online surveys for each of their participating patients before and during the study, which adds extra time to their workloads.

### Sample size

The power analysis indicated that at least 164 patients would need to be included in this study to obtain results indicating a practically relevant improvement in the therapeutic working alliance. The standard deviation (SD) used is based on previous research on the psychometric properties of the Working Alliance Inventory (WAI-12). The power analysis was performed in G*Power version 3.1.9 (27) for the comparison of two independent groups (t-test) with a power of (1-ß) = .80, a significance level <.05, a minimum clinically relevant difference of half a SD = .39, and an allocation ratio of 1. This number of patients is required based on the more realistic result of half, rather than a full SD. For more in-depth and precise analyses using a linear regression analysis, including the interaction variables, 209 patients will be needed. Initially, the investigators aim to enroll 164 patients.

### Measures

#### General questions and demographics

The demographic questionnaire for migrant patients (and confidants) includes the date, preferred language, name, (patient’s name, relationship to patient), mental health center of treatment, age at the time of admission, sex, age at arrival in the Netherlands, and country of origin. For migrant patients who were born in the Netherlands, the country of origin of both parents was included. In addition, the patient’s and parents’ level of education, patient’s occupation, profession, religion, level of religiosity, and previous diagnoses will be recorded. The clinician questionnaire includes the date, name, age, sex, country of origin, mental health center of work, profession, work experience in mental health, and prior experience or training in the use of the CFI.

#### The cultural formulation interview

The 16 semi-structured questions of the CFI serve as the intervention (10). The CFI aims to bridge cultural differences by explicitly asking about someone’s cultural background and its influence on the problems presented. Questions cover the cultural definition of the problem; cultural perceptions of its cause, context, and support; cultural aspects of coping; and past and present help-seeking behaviors. The informant version of the CFI (CFI-I), which will be used in consultations with confidants, consists of 17 questions covering the same topics. Clinicians schedule approximately one hour for the treatment sessions in which they use the CFI questions and context only with the patients in the intervention group. Clinicians are prompted to follow the outline of the CFI and CFI-I as provided in the DSM-5 (10). In the control group, these types of questions need to be avoided, whilst in both groups the necessary care will always be provided.

#### The cultural formulation interview fidelity check

The CFI fidelity check that is provided in the DSM-5 (28) will be used to verify compliance with the CFI questions in the intervention group and abstinence from the CFI questions in the control group. The fidelity check consists of 12 short, yes or no questions. The questions cover topics such as causes of the symptoms, severity of the symptoms, things that improve the symptoms, things that make symptoms worse, coping, identity, and past help-seeking behavior. It takes about five minutes to complete the fidelity check.

#### Working alliance

The main outcome indicating the strength of the therapeutic working alliance will be estimated using the Working Alliance Inventory (WAI) (29). The WAI consists of 36 items for clinicians and 12 items for migrant patients using a 5-point Likert scale. It takes clinicians approximately 10 minutes to complete the WAI online. Completing the WAI in person with the patients takes approximately 15 minutes. Available validated patient versions of the WAI are used in English, Dutch, Spanish, Turkish, and Arabic. In questions one and eleven of the Arabic WAI, the translation for the word indicating “problem” was changed because the Arabic word used in these two questions means “defect”, which has a negative connotation. The meaning of this word was independently checked by two native Arabic speakers, both of whom recommended that the word be changed to “problem”. Interpreters are used when translation into other languages is required, or when none of the researchers or experts by experience speaks the same language as a participating illiterate migrant patient.

#### Clinicians’ level of cultural empathy

The Scale of Ethnocultural Empathy (SEE) will be used to measure the clinician’s level of cultural empathy in the context of the previous treatment session(s) (30). The 31 items are scored on a 6-point Likert scale. For this study, three researchers independently translated the English SEE into Dutch. Differences in the translations were discussed until a consensus was reached. The scale measures four empathy factors: empathic feeling and expression (EFE), ethnocultural empathy awareness (EA), acceptance of cultural differences (AC), and empathic perspective-taking (EP). It takes approximately 10 minutes for clinicians to complete the questionnaire.

#### Perceived empathy

The Barrett-Lennard Relationship Index (BLRI) will be used to measure the perceived empathy of migrant patients and their confidants (31, 32). It was decided to use the validated 12-item version. Items are rated on a 6-point Likert scale. It takes approximately 15 minutes for the researchers to complete the BLRI together with the patients. Available validated versions of the BLRI are used in English, Dutch, Spanish, and Turkish. Interpreters are used when translation into other languages is required. **Table 1** shows an overview of the timing and the type of questionnaires for each participant group.

**Table 1:** Overview of timing and type of questionnaire for each participant group.

After enrollment	After the first treatment	After the fifth treatment	After the consultation with the clinician	After the first treatment	After the fifth treatment
**All Participants**	**Patient**	**Patient**	**Confidant**	**Clinician**	**Clinician**
**Informed consent**	**BLRI-12**	**BLFI-12**	**BLRI-12**	**CFI Fidelity Check-12**	**CFI Fidelity Check-12**
**Demographic questions**		**WAI-12**		**SEE-31**	**SEE-31**
					**WAI-36**

Blue indicates the questionnaires for all participants that are administered by the researchers, Red indicates the questionnaires of migrant patients, Yellow indicates the questionnaire for confidants, and Orange indicates the questionnaires for clinicians.

### Data collection and management

A data management plan is available online at: https://dmponline.dcc.ac.uk/plans. The Tilburg University Qualtrics portal will be used for data collection. Data analysis, monitoring, and cleaning will be performed during and after the data collection. This process will be monitored by the research team, and the principal investigator will be blinded before the data analysis starts.

### Data analysis

Analyses will be performed using intention-to-treat principles. Missing data will be imputed using multiple imputation if assumptions are not violated. Non-parametric equivalents (Mann-Whitney U-tests) will be used when appropriate. All analyses will be performed taking into account the structure of the dataset (individuals within the four institutions: migrant patients, confidants, and clinicians). Data will be coded according to randomization.

**Figure 2:**
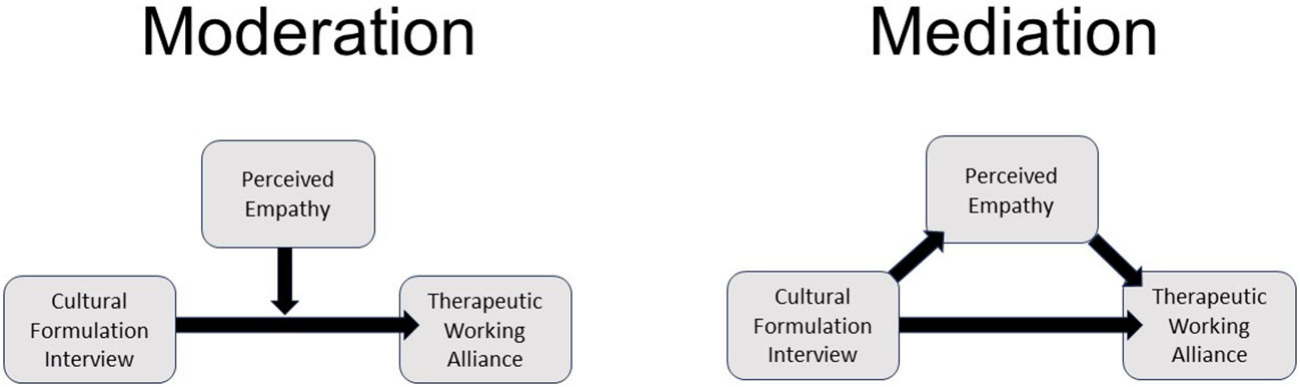
Perceived Cultural Empathy as Moderation or Mediation Variable in the Effect of the Cultural Formulation Interview on Therapeutic Working Alliance.

Data analysis will be performed using SPSS-28. SEE, BLRI, and WAI scores will be calculated according to the instrument’s guidelines. Mean WAI scores between the intervention and control groups will be compared using t-test analyses. Linear regression analysis will be used to examine the main effect of CFI use on therapeutic working alliance and to test whether perceived empathy (BLRI and SEE) moderates (changes in strength and direction) the relationship between the use of the CFI and therapeutic working alliance when this relationship occurs. In addition, it will be tested whether perceived empathy (BLRI and SEE) mediates (explains) the relationship between the use of the CFI and working alliance within the framework of Baron & Kenny (33). **Figure 2** shows the to be examined moderating or mediating effect of perceived empathy in the analyses. To test for differences in the three analyses above, age, sex, generational status, country of origin, educational level, occupation, profession, religion, and religiosity of the migrant patients will be included as covariates or grouping variables in the analyses. In addition, the influence of gender differences among clinicians, work experience, and prior experience with the CFI will be explored.

### Quality assessment

First, the quality of this study will be ensured by training all participating clinicians in the use of the CFI and by organizing evaluations and fidelity checks during the study. This will increase the clinicians’ competence in using the CFI and their insight into the differences between treatment sessions with and without the CFI. Knowing and experiencing these differences will help them adhere to the study protocol while ensuring the quality of treatment and care for migrant patients in both the intervention and control groups. There was some debate about providing instructions for the content of the standard questions in the control group (34). It was decided not to provide any because it would have meant changing the study protocol by creating two intervention groups with different interventions, rather than one intervention and one control group as originally planned. With the agreement of all researchers, the study protocol remained unchanged, and no instructions will be provided on how to omit the CFI questions in the control group to ensure treatment as usual. The questions in the CFI-Fidelity check will monitor the treatment quality in both groups, based on variations in the answers.

## Discussion

Based on what has been described in the literature, this is the first RCT with the CFI. This study aims to assess the effectiveness of the CFI on therapeutic working alliances, including the influence of cultural empathy, and to confirm the benefit of its use in mental health care for migrant patients. Positive findings may indicate the general implementation of the CFI in clinical practice. They may ultimately contribute to better treatment outcomes and compliance for migrant patients. This study is important because it may help to convince health insurers to support implementation. The results may motivate clinicians to participate in CFI training and improve their transcultural competencies, treatment, and care. Most importantly, positive results will be a step forward in improving mental health care for the many migrant patients who need this care in a safe and understanding environment.

### Strengths and limitations

The first strength of this study is that it is the first study to investigate the effect of the CFI on therapeutic working alliances involving four mental health care institutions with a wide variety of migrant patients. Second, the level of clinician experience with the CFI varies widely across the participating institutions, providing an opportunity to assess its utility in the light of many different circumstances that best represent Dutch clinical practice at this time. A third strength of this study is the added value of the involvement of experts by experience. Their input may increase the clinical significance of this study based on their experience, knowledge, and ideas about mental health care for the included patients. A fourth strength of this study may be the clinical relevance and importance of the research question, and thus, if significant differences in outcome measures are found, the potential to support the overall implementation of the CFI in clinical practice. Finally, to avoid counterproductive use of the CFI, all clinicians will be trained and their experience will be monitored and evaluated. This may reduce the risk of jeopardizing therapeutic working alliances due to incompetent use of, and lack of understanding of the context of the CFI (7).

The first limitation to consider regarding this multicenter RCT is its complexity. Many different parties are involved, especially during recruitment and data collection, which must be carefully managed and monitored throughout the process. Any ambiguity in roles can delay progress, complicate enrollment, and possibly lead to participant withdrawal. In addition, the variety of languages spoken by patients can cause problems in explaining the study, answering questions, and completing the questionnaires. In an attempt to overcome language barriers, precautions were taken by providing information letters in several different languages, using validated translations of the questionnaires, and using licensed interpreters when and where needed. Another limitation may be the large sample size needed to obtain reliable results while participants are free to stop participating and may drop out for many reasons. This aspect may compromise the power of this study. To reduce this risk, rather than stopping for this reason, and to be able to include sufficient migrant patients, more mental health centers could be included. Finally, the CFI training given to all participating clinicians and any prior experience of clinicians working with the CFI before participation may influence and prime the content of the conversations in the control group, despite efforts to avoid using CFI questions in the control group. The decision to conduct this study pragmatically may therefore lead to contamination and underestimation of the results.

### Ethics and dissemination

Information about this study will be provided in writing in several languages, including Dutch, English, Spanish, French, Turkish, Arabic, and Farsi. The content of these letters will also be recorded and made available online on a website specifically designed for this study: Samen Sterker Studie. This website provides brief general information, as well as photographs of the research team members, contact information, and the languages spoken by the principal investigator and the experts by experience. All communication will emphasize the voluntary nature of participation and the fact that all necessary care will be provided. Data are anonymized before publication.

To obtain informed consent and data from migrant patients, personal appointments will be made to clarify information and ensure that they understand the information and the commitment required to participate. Data will be collected from migrant patients in person or online via TEAMS. Valid translations of the BLRI into Dutch, English, Spanish, and Turkish, and of the WAI into Dutch, English, Spanish, Turkish, and Arabic will be used. In situations where other translations are required, licensed interpreters will be consulted.

The results will be disseminated to interested patients who have explicitly asked for insight into the outcomes, clinicians, mental health centers, and patient associations with the help of experts by experience. In addition, results will be presented at relevant national and international conferences or symposia. An article will be submitted for publication in a national and international journal.

## Data availability statement

The raw data supporting the conclusions of this article will be made available by the authors, without undue reservation.

## Ethics statement

The studies involving humans were approved by the ethical review board of the Tilburg Law School. The studies were conducted in accordance with the local legislation and institutional requirements. The participants provided their written informed consent to participate in this study.

## Author contributions

AB: Conceptualization, Methodology, Writing – original draft, Writing – review & editing. SPG: Conceptualization, Writing – review & editing. ND: Conceptualization, Writing – review & editing. HJ: Conceptualization, Methodology, Writing – review & editing. SG: Conceptualization, Writing – review & editing. BS: Conceptualization, Writing – review & editing. HV: Conceptualization, Writing – review & editing. KH: Conceptualization, Writing – review & editing. ÖB: Conceptualization, Writing – review & editing. DA: Conceptualization, Writing – review & editing. MB: Conceptualization, Funding acquisition, Supervision, Writing – review & editing.
